# Expression profiling of long noncoding RNA identifies lnc‐MMP3‐1 as a prognostic biomarker in external auditory canal squamous cell carcinoma

**DOI:** 10.1002/cam4.1213

**Published:** 2017-09-29

**Authors:** Hong Liu, Chunfu Dai, Qianru Wu, Hongyan Liu, Feitian Li

**Affiliations:** ^1^ Department of Otology and Skull Base Surgery Hearing Research Key Lab of Health Ministry of China Eye & Ear Nose and Throat Hospital Fudan University Shanghai China

**Keywords:** Bioinformatics, external auditory canal squamous cell carcinoma, gene expression assay, lncRNA, outcome

## Abstract

Our previous studies suggested external auditory canal squamous cell carcinoma (EACSCC) is a rare malignancy with heterogeneous outcomes. This study aimed to identify lncRNA profile of EACSCC and determine the clinical application. Differential expression genes (DEGs) were investigated in EACSCC by whole transcriptome lncRNA arrays (GPL23178). RT‐PCR was used to quantify the microarray data. Bioinformatics analyses were performed to evaluate DEGs regulations in gene ontology and cellular pathways. Fluorescence in situ hybridization (FISH) was utilized to validate lncRNA expression. The overall survival was determined by Kaplan–Meier and log‐rank analyses. Our microarrays data had been submitted to Gene Expression Omnibus (GSE98912). We identified 5621 DEGs (3185 mRNAs, 2436 lncRNAs) in EACSCC. Lnc‐MMP3‐1 was the top one upregulated lncRNA in EACSCC with fold change of 237.2 (*P* < 0.001). RT‐PCR results showed similar expression levels as microarrays data. Bioinformatics analyses indicated development of EACSCC was involved in aberrant alternations of multiple biological processes and cellular pathways. FSIH assays also found lnc‐MMP3‐1 was significantly differentially overexpressed in EACSCC (*P* < 0.001). Tumor lnc‐MMP3‐1 levels were closely associated with differentiation degree (*P* = 0.016), tumor invasion (*P* = 0.015) and TNM stage (*P* = 0.015). Moreover, lnc‐MMP3‐1 expression was a significant prognostic factor in EACSCC (*χ* ^2^ = 4.276, *P* = 0.039). The study is the first screening and analysis of lncRNAs profile in EACSCC and provides new insights into pathogenesis of this rare disease. Our findings offered convincing evidences that lnc‐MMP3‐1 is a novel survival predictor of EACSCC patients.

## Introduction

External auditory canal squamous cell carcinoma (EACSCC) is a rare but fatal malignancy. The incidence of EACSCC is increasing worldwide and this trend will continue over the next decade 1. The understanding of tumor biology and quality of surgery have been developed vigorously. But EACSCC is benefited less than other malignant neoplasms from the advances in the field of oncology. Based on our previous serial studies, the 2‐year overall survival rate was 56.9% totally, 100% for stage I–III, and 22.3% for stage IV 2, 3, 4, 5, 6, 7, 8, 9. The prognosis is very poor in the absence of appropriate intervention. The diagnosis and management of EACSCC are challenging due to lack of specific clinical manifestations during early stage of the disease. Furthermore, no specific biomarker has been accepted and used routinely in EACSCC until now. Therefore, the carcinogenesis and progression of EACSCC need to be further explored urgently.

Mountainous studies have attempted to the development mechanisms of squamous cell carcinoma of the head and neck (SCCHN). But, the understanding of cellular biology in EACSCC is not as well defined as in other SCCHNs, such as nasopharyngeal carcinoma or laryngeal squamous cell carcinoma 10. While the specialties of EACSCC are often ignored the cellular biology of EACSCC. The significant difference of EACSCC from other cutaneous tumors in head and neck were identified as follows. (1) For other cutaneous tumors, basal cell carcinoma is about four times more common than SCC, while this ratio is reversed in EAC 11. (2) Malignant transformation from benign papillomatosis in EAC could be seen 12. (3) The histopathological differences also exist between EACSCC and other cutaneous tumors 13. (4) EACSCC is more biologically aggressive, showing a high frequency of tumor invasion, lymph node metastasis, and local recurrence 2, 3, 4, 5, 6, 7, 8, 9. So, EACSCC is an interesting entity which should be explored.

Recently, long noncoding RNAs (lncRNAs), more than 200 nt in length, have been drawn more attentions in cancer research. Considerable evidences show that lncRNAs have a myriad of functions in different cellular processes, including cell cycle distribution, differentiation, proliferation, and apoptosis 14, 15, 16. LncRNAs are pervasively transcribed and involved in tumor progression, chemosensitivity, and prognosis of many cancers, such as gastric cancer, lung cancer, renal cancer, or breast cancer 16, 17, 18. However, to our knowledge, little is known about lncRNAs expression profiles in EACSCC. The potential clinicopathological significance of lncRNAs in EACSCC has not been reported until now.

In the present study, we screened gene expression profiles in eight pairs of EACSCC tissues and normal external auditory canal epithelium (EACE) by whole transcriptome lncRNA arrays (GSE98912). Profiling data had been submitted into Gene Expression Omnibus (GEO) (https://www.ncbi.nlm.nih.gov/geo/query/acc.cgi?acc=GSE98912). Using this data, we further validated the expression of selected lncRNAs and mRNAs and conducted integrated bioinformatics analyses. This study was aimed to provide in‐depth understanding of lncRNA in carcinogenesis and identify clinically relevant targets in EACSCC.

## Methods and Materials

### Patients and tissues

Eight patients with EACSCC were collected in our hospital from June 2015 to March 2016. EACSCC was confirmed by histopathological examination. Patients did not receive chemotherapy or radiotherapy preoperatively. All medical documents and pathological specimens were reviewed. The Pittsburgh staging system as modified by Moody et al. 19. was used to restage EACSCC pathologically. One pair of samples was collected from surgical specimen for each case: EACSCC and corresponding normal EACE tissue. The EACE was sampled at a distance of more than 2 cm from tumor margin. Light microscope examination suggested that EACE was free of degeneration and inflammation.

A validation cohort of EACSCC patients treated with surgery was also selected in our hospital from June 2013 to July 2016. Detail operation procedure was reported in our previous studies 2, 3, 4, 5, 6, 7, 8. A part of those patients also had been analyzed in our previous serial studies 4, 5, 6. Patients selection and tissue collection were described above. Each sample was stored in RNA Fixer Reagent (Bioteke, Beijing, China) at 80°C until use.

### LncRNA expression analysis

Experimental processes had been summarized to GEO (GSE98912). Briefly, total RNA was extracted, amplified, labeled, and purified following the manufacturer's instructions. OE whole transcriptome lncRNA arrays (GPL23178) were used to detect expression profile of mRNA and lncRNA (Affymetrix, Santa Clara, CA, US). Slides were scanned by GeneChip^®^ Scanner 3000 and Command Console Software 4.0 was used to extract raw data (Affymetrix, Santa Clara, CA, US). Expression Console (version 1.3.1, Affymetrix) software offered Robust multi‐array average (RMA) normalization of data for both gene and exon level analysis. Then the gene expression analyses were proceeded by Genesrping software (version 13.0; Agilent Technologies).

### RT‐PCR

RNA extraction, reverse transcription, and amplification were performed according to the manufacturer's instructions. Gene expression levels were calculated using the threshold cycle (*C*_t_) method and GAPDH was set as reference. Δ*C*_t_ = *C*_t_ gene−*C*_t_ GAPDH; ΔΔ*C*_t_ = (C_t_ gene−*C*_t_ GAPDH) sample‐ (*C*_t_ gene−*C*_t_ GAPDH) normalization. All primers were synthesized by Oebiotech (Shanghai, China). The sequence of primers was shown in Table S1. PCR cycling conditions were as follows: 94°C for 3 min per cycle, followed by 30 cycles of 94°C for 30 sec, 57°C for 30 sec and 72°C for 60 sec. All the samples were assayed in triplicate.

### Bioinformatics analyses

Gene Ontology (GO) analyses (http://www.geneontology.org) were applied to analyze the main function of the differential expression genes (DEGs). PANTHER overrepresentation test was used to calculate the fold enrichment and *P*‐value. False discovery rate (FDR) was calculated to correct the *P*‐value. Cellular signal pathways were identified according to Kyoto Encyclopedia of Genes and Genomes (KEGG). Pathway enrichment analyses were carried out based on Database for Annotation, Visualization and Integrated Discovery (DAVID) 6.8 (http://david.ncifcrf.gov/) database. The threshold of significance was still defined by *P*‐value and FDR. Protein protein interaction (PPI) was analyzed using STRING database (http://www.string-db.org) to find out the significant PPI of DEGs.

### Fluorescence in situ hybridization (FISH)

LncRNA expression was evaluated by FISH using RiboTM Fluorescent In Situ Hybridization Kit (RiboBio, Guangzhou, China) following the manufacturer's instructions. Briefly, the 6‐*μ*m‐thick sections of fresh tissues were blocked with prehybridization buffer at 37°C for 60 min after washing and fixation. The section was incubated with 20 *μ*mol/L lncRNA FISH Probe Mix at 37°C overnight. After washing, FISH preparations were counterstained with DAPI observed in confocal microscopy for appropriate fluorescence filter sets (Leica, Wetzlar, Germany). The lncRNA probe labeled by Cy3 was designed and synthesized by RiboBio Co., Ltd. RiboTM h‐U6 and RiboTM h‐18S were used as a reference control for subcellular localization of lncRNA. The relative expression level of lncRNA was measured through mean fluorodensitometry (MFD) using Leica Qwin V3 image analysis software. The MFD was measured in random 10 nonoverlapping fields using the following formula: MFD = IOD/AOI (IOD: integrated option density; AOI: area of interesting). The FISH assays were replicated three times.

### Statistical analysis

After RMA normalization of raw data, DEGs between EACSCC and EACE were identified using One‐Way Between‐Subject ANOVA. We set a standard threshold for DEGs of fold change >2.0 and *P*‐value < 0.05. The patterns of gene expression among different tissues were analyzed using hierarchical clustering. The relative expression levels of genes were illustrated as heat map. Red color represented an expression level above mean and green color represented expression level lower than mean. Differences of continuous variables among different groups were analyzed using the one‐way analysis of variance. Associations between categorical variables were analyzed using the Fisher's exact test. Survival curves were calculated by the Kaplan–Meier method. The overall survival was calculated from the diagnosis to death. Univariate log‐rank test and Cox regression model analysis were performed to identify prognostic factors. A significant difference was defined as a two‐tailed *P*‐value of less than 0.05. All statistical analysis was performed using SPSS.17.0 software (SPSS, Chicago, IL, USA).

## Results

### Overview of LncRNA profile

**Figure 1:**
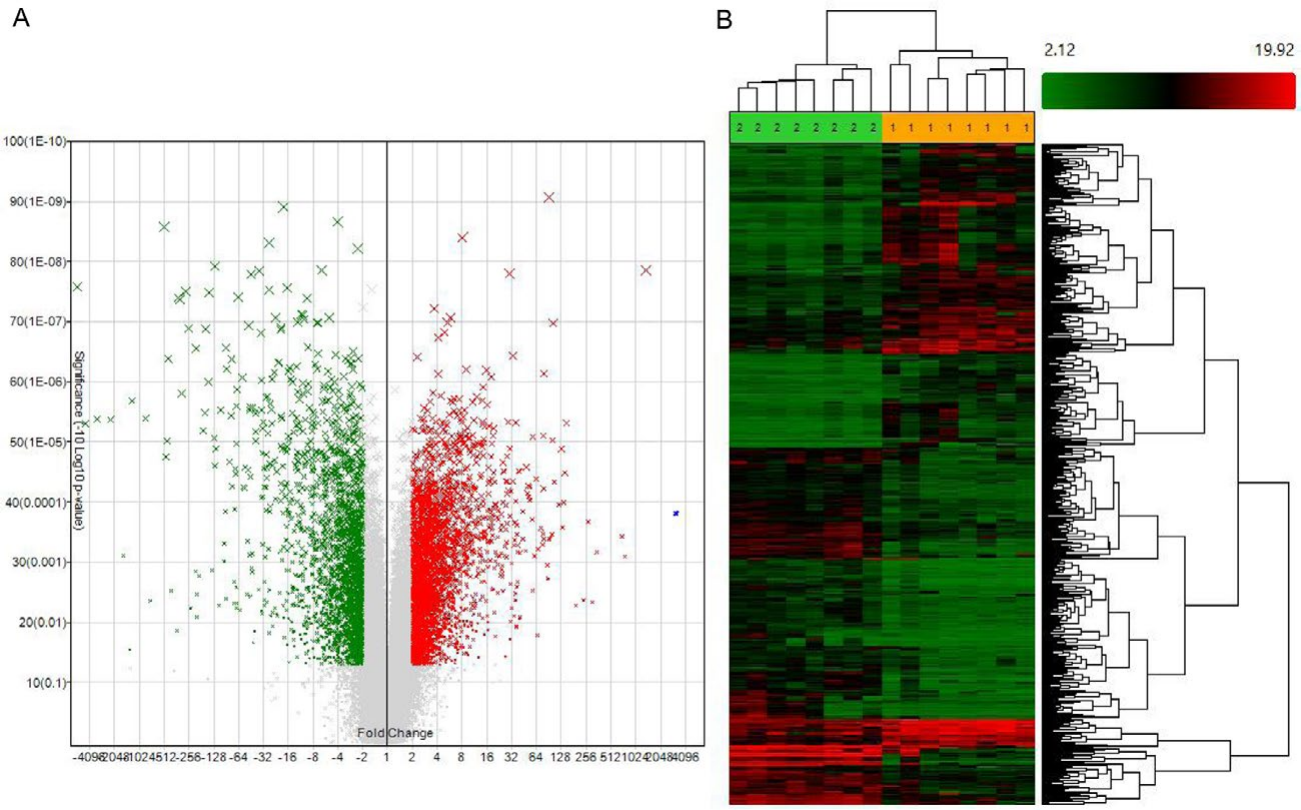
Aberrant expression of genes in external auditory canal squamous cell carcinoma (EACSCC) compared with normal external auditory canal epithelium (EACE) tissues. The volcano plot (A) and hierarchical clustering analyses (B) of dysregulated genes. Red color indicates overexpression and green color indicates low expression. Every column (1:EACSCC, 2:EACE) represents a sample and every row represents a probe.

Microarray data had been submitted to GEO database (GSE98912). The clinicopathological features of the selected eight patients with EACSCC were listed in Table S2. Out of a collection of 90,675 probes (27,133 mRNAs, 63,542 lncRNAs), our whole transcriptome lncRNA arrays identified 5621 DEGs (3185 mRNAs, 2436 lncRNAs). Totally, 3248 genes were upregulated in EACSCC tissues compared with EACE tissues, including 1939 mRNAs and 1309 lncRNAs. While 2373 genes were downregulated in EACSCC tissues compared with EACE tissues, including 1246 mRNAs and 1127 lncRNAs (Fig. 1). As we expected, the genes expression profiles were significantly different between malignant and normal tissues in the external auditory canal.

Hierarchical clustering of the DEGs was performed using Genesrping software. Hierarchical clustering of the expression for DEGs based on Pearson correlation separated EACSCC from EACE tissues, including differentiated mRNAs and lncRNAs. Those differences allowed distinguishing EACSCC and EACE tissues accurately based on the molecular signature (Fig. 1).

### Validation of whole transcriptome lncRNA arrays

**Figure 2:**
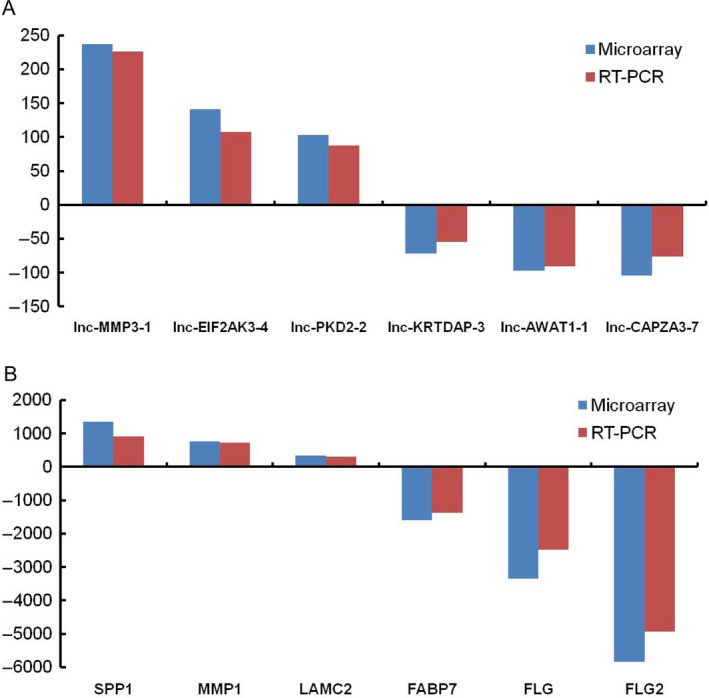
Validation of lncRNAs (A) and mRNAs (B) expression levels by RT‐PCR.

Of those 3248 upregulated DEGs in EACSCC tissues, lncRNA MMP3‐1, EIF2AK3‐4, PKD2‐2, and mRNA SPP1, MMP1, and LAMC2 showed the greatest degree of upregulation, with the fold changes of 237.2, 141.0, 103.3 for lncRNAs and 1360.6, 765.4, 342.5 for mRNAs, respectively. Of those 2373 DEGs that were downregulated in EACSCC tissues, lncRNA KRTDAP‐3, AWAT1‐1, CAPZA3‐7, and mRNA FABP7, FLG, and FLG2 demonstrated the greatest degree of downregulation, with the fold changes of 71.5, 97.0, 104.7 for lncRNAs and 1595.7, 3350.1, 5832.9 for mRNAs, respectively (Fig. 2).

To validate the accuracy of whole transcriptome lncRNA arrays, we detected the expression of DEGs by RT‐PCR assays. We selected the most significant differentiated genes, including six lncRNAs and six mRNAs. When comparing the expression of genes with EACE tissues, lncRNA MMP3‐1, EIF2AK3‐4, PKD2‐2, and mRNA SPP1, MMP1, LAMC2 were found to be upregulated in EACSCC tissues, with 226.0‐, 107.6‐, 88.0‐fold increases for lncRNAs and 910.2‐, 724.1‐, 298.2‐fold increases for mRNAs, respectively. Compared with normal tissues, lncRNA KRTDAP‐3, AWAT1‐1, CAPZA3‐7, and mRNA FABP7, FLG, FLG2 were downregulated in EACSCC tissues, with 54.6‐, 90.5‐, 76.1‐fold decreases for lncRNAs and 1370.0‐, 2486.7‐, 3565.8‐fold decreases for mRNAs, respectively. The expression difference of genes between EACSCC and EACE tissues was significant (*P* < 0.001 for all genes). Furthermore, when expression data from lncRNA arrays were compared with RT‐PCR results, all selected genes showed similar expression levels (Fig. 2).

### Functional annotation and pathway analyses

**Figure 3:**
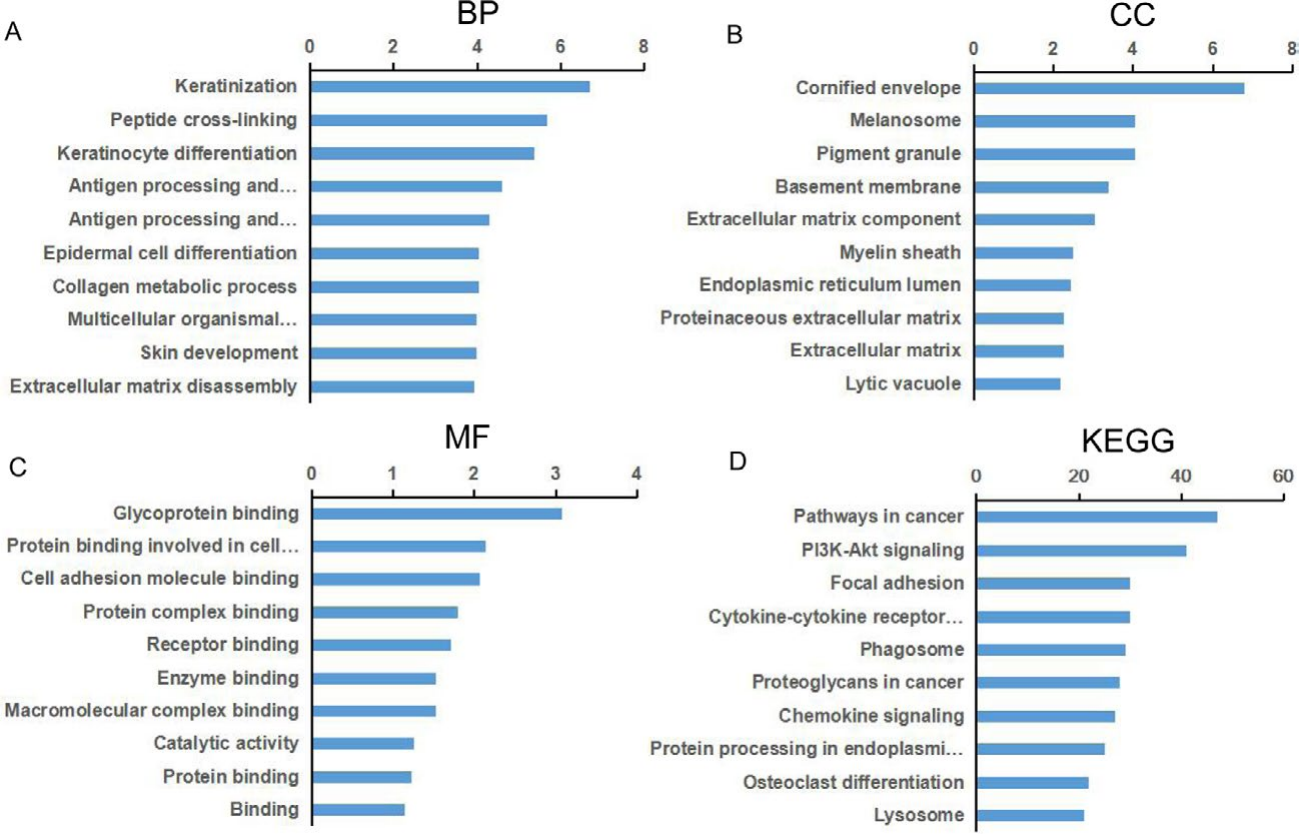
Bioinformatics analyses of differentially expressed genes in external auditory canal squamous cell carcinoma (EACSCC). (A) Top 10 enriched terms of biological process (BP); (B) Top 10 enriched terms of cellular component (CC); (C) Top 10 enriched terms of molecular function (MF); (D) Top 10 enriched biological pathways of differentially expressed genes in SSCC. The vertical axis represents the enriched category and the horizontal axis represents the enrichment of dysregulated genes.

GO enrichment analyses of DEGs were performed related to biological process (BP), cellular component (CC), and molecular function (MF). Top significant terms of BP, CC, and MF were provided in Tables [Link], [Link], [Link], respectively. GO enrichment analyses indicated that these DEGs were enriched in 195 terms of BP. The majority were related to epidermis development‐related biological behaviors and the top three were keratinization, peptide cross‐linking, and keratinocyte differentiation. DEGs were mainly enriched in 79 terms of CC and the top three were cornified envelope, melanosome, and pigment granule. Similarly, 14 terms of MF were proven to be enriched for DEGs and the top three were glycoprotein binding, protein binding involved in cell adhesion, and cell adhesion molecule binding (Fig. 3).

To investigate underlying biological associations, we ran KEGG pathway analyses of DEGs. Top significant pathways were shown in Table S6. The results demonstrated DEGs were enriched in 31 pathways. Enrichment pathways were mainly involved in cancer‐associated functions. The top three pathways with the maximum number of enriched genes were pathways in cancer, PI3K‐Akt signaling pathway and focal adhesion (Fig. 3). PPI network was constructed for DEGs to investigate the global functions (Fig. S1). The network analysis suggested that DEGs interacted with other several genes and regulated by tumor‐associated genes. With the net, we screened the important dysregulated genes between EACSCC and EACE tissues. According to the results of this analysis, DEGs located in the center of PPI network were key genes. The results also showed that the core genes may have played an important role in EACSCC.

### Clinical information of patients in validation cohort

Totally, we collected 43 patients with EACSCC in validation cohort based on inclusion criteria. The clinicopathologic features of those 43 patients were shown in Table 1. The patients included 30 men and 13 women. The ages were ranged from 35 to 79 years with median of 63 years. A total of 25 patients were diagnosed with left‐sided disease. Tumor size varied among those patients (0.06–92.50 cm^3^). According to criteria of WHO tumor differentiated grade, 18 cases in G1, 21 cases in G2 and 4 cases in G3. The majority of patients were T4 (53.5%) and negative lymph node involvement (90.7%). Postoperative pathologic exam revealed 1 patient with stage I, 9 with stage II, 10 with stage III, and 23 with stage IV. Surgery was the first choice of treatment for EACSCC, if the tumor might possibly be completely resected. The decision to use postoperative radiotherapy and/or chemotherapy was individualized for each patient. Detail operation procedure, chemotherapy regimens, or radiotherapy doses were reported in our previous studies 2, 3, 4, 5, 6, 7, 8, 9. The treatment modalities were described in Table S7.

**Table 1:** Clinicopathological features of EAC SCC patients

Variables	Cases (43)	The expression of lnc‐MMP3‐1		*P*‐value
		Low (19)	High (24)	
Age				0.223
≥63 years	22	12	10	
<63 years	21	7	14	
Sex				0.743
Male	30	14	16	
Female	13	5	8	
Laterality				0.756
Left	25	12	13	
Right	18	7	11	
Differentiation				0.016
G1	18	12	6	
G2 + G3	25	7	18	
Tumor invasion				0.015
T1 + T2 + T3	20	13	7	
T4	23	6	17	
Lymph node				0.118
Positive	4	0	4	
Negative	39	19	20	
TNM stage				0.015
I + II + III	20	13	7	
IV	23	6	17	

### Lnc‐MMP3‐1 overexpression in EACSCC

**Figure 4:**
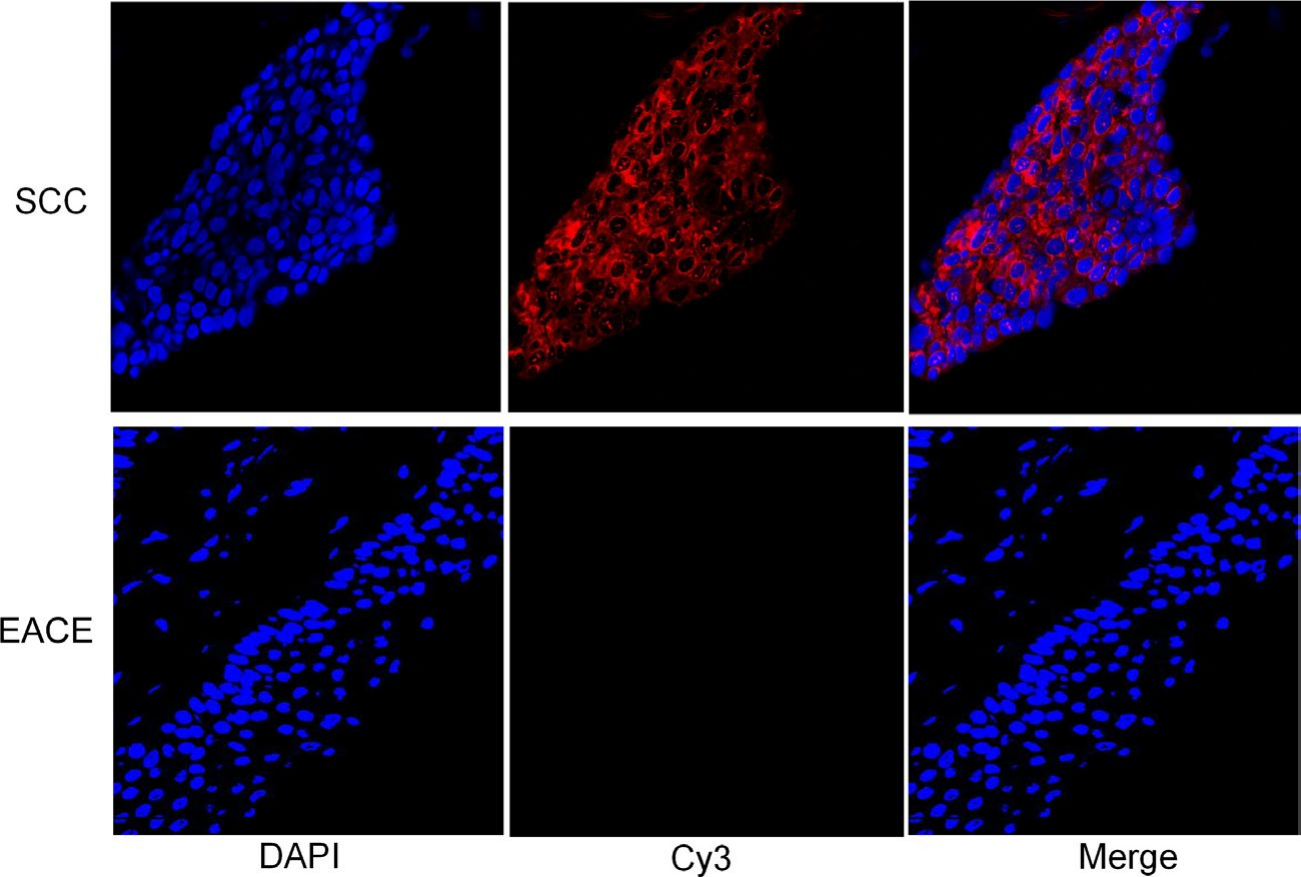
Detection of lnc‐MMP3‐1 expression in external auditory canal squamous cell carcinoma (EACSCC) and normal external auditory canal epithelium (EACE) by FISH assays.

FISH assays indicated lnc‐MMP3‐1 (red hybridization signals) is localized in cytoplasm in EACSCC tissues. EACSCC showed dominant, strongly positive lnc‐MMP3‐1 expression in the cytoplasm of malignant cells, whereas the EACE had weakly or negative expression (Fig. 4). MFD of EACSCC and EACE tissues was 0.964 ± 0.087 and 0.092 ± 0.015, respectively. The statistical results showed that MFD of lnc‐MMP3‐1 in EACSCC was significantly higher than EACE tissues (*P* < 0.001).

### Correlations between lnc‐MMP3‐1 and clinical parameters

Based on MFD of lnc‐MMP3‐1, threshold value of 0.964 was selected as the cutoff value. Patients in validation cohort were thus divided into two groups: lnc‐MMP3‐1 high expression group (MFD ≥ 0.964, 55.8%) and low expression group (MFD < 0.964, 44.2%).

As shown in Table 1, lnc‐MMP3‐1 expression was closely correlated with differentiation degree (*P* = 0.016), tumor invasion (*P* = 0.015) and TNM stage (*P* = 0.015). However, other clinical parameters, such as age (*P* = 0.015), gender (*P* = 0.015), laterality (*P* = 0.015), and lymph node metastasis (*P* = 0.118), were found not to be significantly correlated with lnc‐MMP3‐1 expression in this study.

### Survival of EACSCC patients

**Figure 5:**
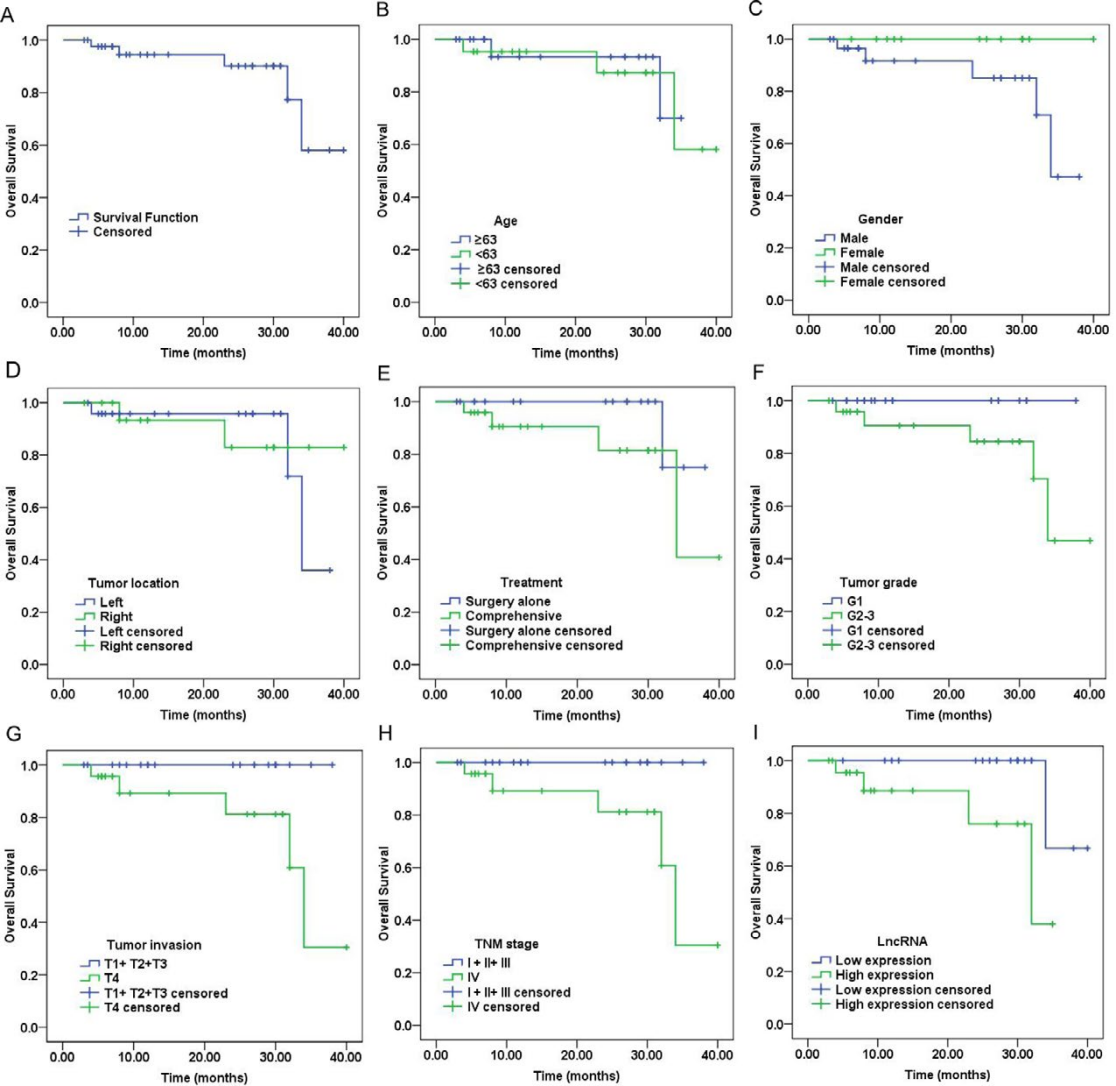
Survival analyses of patients with external auditory canal squamous cell carcinoma (EACSCC). (A): The overall survival of 43 patients with EACSCC; Kaplan–Meier analyses of EACSCC patients according to age (B), gender (C), tumor location (D), treatment (E), tumor grade (F), tumor invasion (G), TNM stage (H), and lnc‐MMP3‐1 expression (I).

The 1‐, 2‐, 3‐year cumulative overall survival rate of those 43 EACSCC patients was 94.5%, 90.2%, and 58.0%, respectively (Fig. 5). Mean survival time was 34.4 months with the 95% confidence interval (CI) of 31.547 to 37.327 months. Kaplan–Meier analysis showed that tumor invasion (*χ* ^2^ = 4.640, *P* = 0.031), TNM stage (*χ* ^2^ = 4.640, *P* = 0.031) and lnc‐MMP3‐1 expression (*χ* ^2^ = 4.276, *P* = 0.039) were significant prognostic factors of overall survival for EACSCC patients (Fig. 5). However, age (*χ* ^2^ = 0.046, *P* = 0.830), sex (*χ* ^2^ = 2.115, *P* = 0.146), laterality (*χ* ^2^ = 0.040, *P* = 0.842), differentiation grade (*χ* ^2^ = 2.406, *P* = 0.121), and treatment (*χ* ^2^ = 1.752, *P* = 0.186) did not reach the statistical significance (Table 2).

**Table 2:** Survival analyses of EAC SCC patients

Characteristics	Survival analysis	
	*χ* ^2^	*P*‐value
Age		
≥63 years versus <63 years	0.046	0.830
Sex		
Male versus female	2.115	0.146
Laterality		
Left versus right	0.040	0.842
Differentiation		
G1 versus G2 + G3	2.406	0.121
Tumor invasion		
T1 + T2 + T3 versus T4	4.640	0.031
TNM stage		
I + II+ III VS. IV	4.640	0.031
Treatment		
Surgery alone versus comprehensive treatment	1.752	0.186
Expression of lnc‐MMP3‐1		
Low versus high	4.276	0.039

We also assessed the correlation between lnc‐MMP3‐1 expression and clinical outcomes. The 1‐, 2‐, 3‐year cumulative overall survival rate of patients with lnc‐MMP3‐1 overexpression was 88.6%, 76.0%, 38.0%, respectively. The 1‐, 2‐, 3‐year cumulative overall survival rate of patients with lnc‐MMP3‐1 low expression was 100.0%, 100.0%, 66.7%, respectively (Fig. 5). The results of Kaplan–Meier analysis showed that the median overall survival time was 29.0 (95% CI: 24.3–33.9) and 38.0 (95% CI: 34.8–41.2) months for EACSCC patients with high and low expression of lnc‐MMP3‐1, respectively.

## Discussion

The experience on diagnosis and management of EACSCC is limited because of its rarity 9. Traditionally, EACSCC is analyzed as a part of SCCHNs and its specialties acquire little attention. Most studies of EACSCC are retrospective review study and focus on the clinical variables analyses. To date, the pathogenesis of EACSCC remains very poorly elucidated from the molecular perspective. Only a few studies focus on its potential molecular targets by immunohistochemistry 20, 21. Recently, increasing evidences showed that carcinogenesis of various cancers are closely associated with abnormal expression of lncRNAs 16, 17, 18, 22, 23. So far, no study attempts to reveal the significance of lncRNA expression in EACSCC to the best of our knowledge.

In the present study, we investigated the lncRNA and mRNA expression profiles of EACSCC using whole transcriptome lncRNA arrays (GPL23178). The microarray data were available in GEO (GSE98912). As shown in Figure 1, we identified 5621 DEGs including 3185 mRNAs, and 2436 lncRNAs in EACSCC compared to EACE. False positive results might exist. So, we selected the top 12 DEGs including lncRNAs and mRNAs to validate the microarray results. RT‐PCR results were consistent with the microarray detection (Fig. 2). Our results demonstrate that those DEGs identified by microarray may act as novel biomarkers for this rare disease.

GO and KEGG pathway analyses were utilized to identify biological functions of DEGs. We found that these DEGs were involved in a lot of cancer‐associated biological processes, such as keratinocyte differentiation, antigen processing, cornified envelope, extracellular matrix component, and cell adhesion (Tables [Link], [Link], [Link]). The functions of DEGs were mainly related to epithelium differentiation, extracellular matrix, cell adhesion, and immune. Go analyses suggested EACSCC development was the process of multigene participation and multistep evolution. The pathogenesis was not only related to the malignant transformation of epithelial cells, but also the reconstruction of extracellular matrix and patients’ immunity. Those findings also demonstrated the complex mechanisms in occurrence of EACSCC. KEGG analyses identified that many pathways enriched by DEGs were mainly related to cancer, such as pathways in cancer, PI3K‐Akt signaling pathway and focal adhesion (Table S6). Pathway in cancer is the most famous signaling pathway involved in the development of human cancers. It has been documented that aberrant alternations of pathways in cancer exist in many cancers 24, 25. PI3K‐Akt signaling pathway regulates fundamental cellular functions, including proliferation, metabolism, cell cycle, and apoptosis 24. Malfunction of PI3K‐Akt signaling pathway could result in uncontrolled cell proliferation and degeneration in lung, stomach, and esophagus 26. Focal adhesion emphasizing cross talk between cells and extracellular matrix is involved in morphological alterations and gene expression modulation 27. Focal adhesion is well‐studied to play essential roles in cancer progression and metastasis 24, 27. Our results demonstrated DEGs were enriched in classical biological processes and pathways, suggesting that DEGs identified by our microarrays are not only differentially expressed, but also have important functions of tumorigenesis and tumor progression in EACSCC. The global PPI network was established to show how DEGs participate in the pathogenesis of EACSCC, although the clinical significance largely remains unknown. PPI network exhibited the betweenness centrality of DEGs and revealed that the core genes play a critical role in EACSCC development (Fig. S1). Thus, our preliminary data provide a rationale for the involvement of DEGs in EACSCC.

According to our data of microarray and RT‐PCR, we identified lnc‐MMP3‐1 with the most significantly differentiated expression as the target gene in our study. Lnc‐MMP3‐1 is located in chr11:102733476‐102745764 (hg19). The size of lnc‐MMP3‐1 was 1865 bp with 11 extrons. Several database were used to evaluate the protein coding ability of lnc‐MMP3‐1, including Proteomics Identifications Database (PRIDE) reprocessing 2.0 28, Lee translation initiation sites 29, PhyloCSF score 30, and BaEEni small ORFs 31. Results suggested lnc‐MMP3‐1 does not code any protein and belongs to noncoding RNA 28, 29, 30, 31. Although the sequence of lnc‐MMP3‐1 had been identified, the biological function of lnc‐MMP3‐1 remains unknown.

In the present study, we assessed lnc‐MMP3‐1 expression profile in 43 patients with EACSCC. We first found that expression of lnc‐MMP3‐1 was increased in ESCC tissues compared with EACE by FISH. Our findings were also consistent with those of LncATLAS database 32, which demonstrated cytoplasmic localization of lnc‐MMP3‐1. For further exploring the clinicopathological roles and prognostic value of lnc‐MMP3‐1, we divided the EACSCC patients into two groups: high expression group and low expression group. Previous studies defined lncRNA expression levels through various classification systems, but there was no common accepted classification system. In this study, we set the median expression value as the cutoff score based on FISH analyses. Therefore, there were 24 EACSCC patients in high lnc‐MMP3‐1 expression level group and 19 patients in low lnc‐MMP3‐1 expression level group. Our data demonstrated that lnc‐MMP3‐1 overexpression was significantly associated with low differentiation degree, tumor invasion, and advanced TNM stage (Table 1). Our results were consistent with previous reports 17, 18, 22, which demonstrated that Ku80 expression was related to important clinicopathological characteristics in esophageal cancer and gastric cancer. These findings suggested that lnc‐MMP3‐1 plays important roles in tumor progression and might be a biomarker for EACSCC patients.

Until now, only a few literature reported biomarkers of EACSCC 20, 21. Liu et al. 20. found that MMP9 and CD34 may play an important role in the invasion and metastasis of EACSCC. Okado et al. 21. demonstrated laminin 5‐*γ*2 could be used as an indicator of prognosis in EACSCC patients. However, no information is available about the effects of lncRNA expression in prognosis of EACSCC patients. One interesting question attracted our attention: whether lnc‐MMP3‐1 could be exploited as a new prognostic biomarker in EACSCC. Here, our data indicated that longer survival of EACSCC patients was related to limited tumor invasion, early TNM stage and low lnc‐MMP3‐1 expression (Table 2). Therefore, assessment of lnc‐MMP3‐1 expression in EACSCC might provide valuable information about clinical outcomes and follow‐up management.

Although, this is the first study to analyze lncRNA expression profile in EACSCC, we acknowledge the limitations of this study. One of the limitations is that sample size is relatively small due to the rarity of this disease. Our findings require further validation in larger cohorts. Another limitation of the study is that we did not perform cellular experiment and lnc‐MMP3‐1 functional analysis. Currently, there is no universal and common accepted specified cell line in EACSCC. So, we just analyzed the clinical significance of lnc‐MMP3‐1. Further studies are required to clarify the mechanism of molecular functions and possible new therapeutic potential of lnc‐MMP3‐1 in EACSCC, both in vitro and in vivo.

In conclusion, we screened the lncRNA expression in EACSCC and showed that DEGs were involved in cancer pathways as a proof of principle. These findings provide new insights into molecular pathogenesis of this refractory malignancy. We also demonstrated that overexpression of lnc‐MMP3‐1 was pronouncedly associated with several adverse clinicopathological features and poor prognosis in EACSCC. Our data suggested that lnc‐MMP3‐1 could be exploited as a new prognostic biomarker for EACSCC patients. These findings lay the foundation and offer valuable clues for future function and mechanism investigation of EACSCC.

## Ethics Statement

This study was approved by the institutional review board of Eye, Ear, Nose, and Throat Hospital affiliated with Fudan University (Shanghai, China). This study protocol meets the Health Insurance Portability and Accountability Act compliance standards. All participants were provided with written, informed consent.

## Conflict of Interest

The authors declare that they have no conflicts of interest.

## Supporting information


**Figure S1.** Protein–protein interaction of dysregulated genes in external auditory canal squamous cell carcinoma (EACSCC) compared with normal external auditory canal epithelium (EACE) tissues.

**Table S1**. The sequence of RT‐PCR primers.

**Table S2**. Clinicopathological features of the selected 8 EAC SCC patients.

**Table S3**. Biological processes enrichment analyses of differential expression genes.

**Table S4.** Cellular components enrichment analyses of differential expression genes.

**Table S5**. Molecular functions enrichment analyses of differential expression genes.

**Table S6.** KEGG enrichment analyses of differential expression genes.

**Table S7.** Treatment modality of EAC SCC patients.
