# Supplementary material for: Expression profiling of long noncoding RNA identifies lnc‐MMP3‐1 as a prognostic biomarker in external auditory canal squamous cell carcinoma
Source: Cancer Med. 2017 Sep 29;6(11):2541–51. doi: 10.1002/cam4.1213 (PMC5673923; doi:10.1002/cam4.1213)
Supplement: Supplementary file 1 — Figure S1. Protein–protein interaction of dysregulated genes in external auditory canal squamous cell carcinoma (EACSCC) compared with normal external auditory canal epithelium (EACE) tissues. [file CAM4-6-2541-s001.pdf]

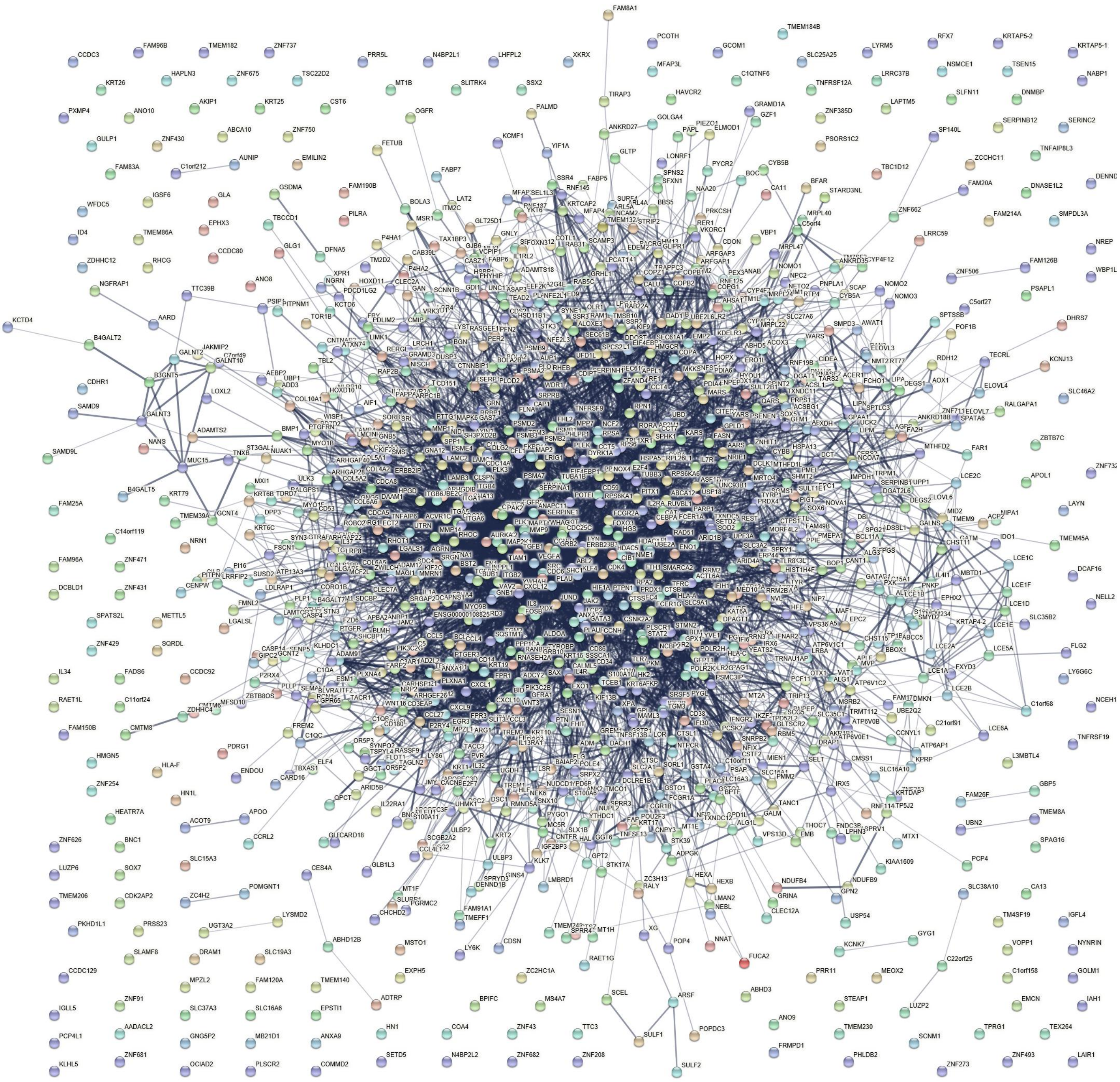

SuppInfo Figure 1. Protein-protein interaction of dysregulated genes in external auditory canal squamous cell carcinoma (EACSCC) compared with normal external auditory canal epithelium (EACE) tissues.
