# Supplementary material for: Expression profiling of long noncoding RNA identifies lnc‐MMP3‐1 as a prognostic biomarker in external auditory canal squamous cell carcinoma
Source: Cancer Med. 2017 Sep 29;6(11):2541–51. doi: 10.1002/cam4.1213 (PMC5673923; doi:10.1002/cam4.1213)
Supplement: Supplementary file 2 — Table S1. The sequence of RT‐PCR primers. [file CAM4-6-2541-s002.doc]

**Supporting Table 1.** The sequence of RT-PCR primers

| Gene | Foreword | Reverse |
| --- | --- | --- |
| lnc-MMP3-1 | CAGCTCTCTGTGACCCCAAT | TCTCTCAGAAACCTTCAGCCA |
| lnc-EIF2AK3-4 | CGGAACCAGACGATGAGACA | GGCAGCAATTCTCCCATCTA |
| lnc-PKD2-2 | CTGGAAGTTCTGAGGAAAAGCA | AGTCAATGGAGTCCTGGCTG |
| lnc-KRTDAP-3 | GAAGATCCCGGTCCTTCCT | CGGGTCGTGACGCATAAT |
| lnc-AWAT1-1 | GGAACTGGTGTGTCTGGACC | GGTGAACCCCCATGAGGTA |
| lnc-CAPZA3-7 | CAGCAATACGAGTCTGGCCT | GTGCTCCAGATCTTCCCAGA |
| SPP1 | CAGCTGTTTTTAACCCACGTTT | TTGGGATAACCAGGGTCCAT |
| MMP1 | ATCACTTCTCCCCGAATCGT | CACAGCTTTCCTCCACTGCT |
| LAMC2 | AGAGAAAGGGACCGCTGTTT | CTGGCTCCTGTCACACCTG |
| FABP7 | GAAATGGGATGGCAAAGAAAC | TGCCTTCTCATAGTGGCGAA |
| FLG | TGTCTCATCCTCATTCAGGTGTT | CTTGGCTCCAAGTTGTCTGG |
| FLG2 | CCTTCAACTTCTGGGCTCAA | AGTTCCAAAAGCTGGCCTTC |
| GAPDH | GGAAGGTGAAGGTCGGAGTC | TGAGGTCAATGAAGGGGTCA |

MMP: matrix metallopeptidase; EIF2AK3: encoding translation initiation factor 2-alpha kinase 3; PKD2: polycystic kidney disease 2; KRTDAP: keratinocyte differentiation-associated protein; AWAT1: acyl-CoA wax alcohol acyltransferase 1; CAPZA3: capping protein (actin filament) muscle Z-line, alpha 3; SPP1: secreted phosphoprotein 1; LAMC2: laminin, gamma 2; FABP7: fatty acid binding protein 7; FLG: filaggrin; FLG2: filaggrin family member 2; GAPDH: glyceraldehyde-3 phosphate dehydrogenase.
