# Supplementary material for: Expression profiling of long noncoding RNA identifies lnc‐MMP3‐1 as a prognostic biomarker in external auditory canal squamous cell carcinoma
Source: Cancer Med. 2017 Sep 29;6(11):2541–51. doi: 10.1002/cam4.1213 (PMC5673923; doi:10.1002/cam4.1213)
Supplement: Supplementary file 3 — Table S2. Clinicopathological features of the selected 8 EAC SCC patients. [file CAM4-6-2541-s003.doc]

**Supporting Table 2.** Clinicopathological features of the selected 8 EAC SCC patients.

| Cases | Age | Sex | Laterality | TNM  stage | Tumor size (cm^3^) | Differentiation |
| --- | --- | --- | --- | --- | --- | --- |
| 1 | 51 | female | right | T2N0M0, Ⅱ | 1.0×0.6×0.3 | Ⅱ |
| 2 | 52 | male | left | T4N0M0, Ⅳ | 5.5×4.0×3.5 | Ⅱ |
| 3 | 79 | male | left | T4N0M0, Ⅳ | 1.5×1.2×0.5 | Ⅱ |
| 4 | 74 | male | right | T3N0M0, Ⅲ | 0.9×0.5×0.5 | Ⅰ |
| 5 | 60 | female | right | T3N0M0, Ⅲ | 1.0×0.9×0.5 | Ⅰ |
| 6 | 62 | female | left | T4N0M0, Ⅳ | 4.5×2.2×1.5 | Ⅰ |
| 7 | 35 | female | right | T2N0M0, Ⅱ | 1.5×0.5×0.4 | Ⅰ |
| 8 | 63 | male | right | T3N0M0, Ⅲ | 1.0×0.8×0.5 | Ⅰ |
