# Supplementary material for: Expression profiling of long noncoding RNA identifies lnc‐MMP3‐1 as a prognostic biomarker in external auditory canal squamous cell carcinoma
Source: Cancer Med. 2017 Sep 29;6(11):2541–51. doi: 10.1002/cam4.1213 (PMC5673923; doi:10.1002/cam4.1213)
Supplement: Supplementary file 4 — Table S3. Biological processes enrichment analyses of differential expression genes. [file CAM4-6-2541-s004.doc]

**SuppInfo Table 3**. Biological processes enrichment analyses of differential expression genes

| GO ID | Term | Gene | Fold Enrichment | P value |
| --- | --- | --- | --- | --- |
| 0031424 | keratinization | 21 | 6.68 | 2.01E-07 |
| 0018149 | peptide cross-linking | 20 | 5.68 | 9.33E-06 |
| 0030216 | keratinocyte differentiation | 34 | 5.36 | 8.77E-11 |
| 0042590 | antigen processing and presentation of exogenous peptide antigen via MHC class I | 19 | 4.58 | 6.85E-04 |
| 0002479 | antigen processing and presentation of exogenous peptide antigen via MHC class I, TAP-dependent | 17 | 4.29 | 7.72E-03 |
| 0009913 | epidermal cell differentiation | 37 | 4.03 | 2.54E-08 |
| 0032963 | collagen metabolic process | 19 | 4.03 | 4.60E-03 |
| 0044259 | multicellular organismal macromolecule metabolic process | 20 | 3.98 | 2.87E-03 |
| 0043588 | skin development | 53 | 3.98 | 9.30E-13 |
| 0022617 | extracellular matrix disassembly | 19 | 3.93 | 6.75E-03 |
| 0008544 | epidermis development | 58 | 3.58 | 3.38E-12 |
| 0044236 | multicellular organism metabolic process | 20 | 3.35 | 3.72E-02 |
| 0006665 | sphingolipid metabolic process | 31 | 3.33 | 1.15E-04 |
| 0034620 | cellular response to unfolded protein | 23 | 3.18 | 1.73E-02 |
| 0030968 | endoplasmic reticulum unfolded protein response | 22 | 3.13 | 3.77E-02 |
| 0035967 | cellular response to topologically incorrect protein | 24 | 2.98 | 3.08E-02 |
| 0002449 | lymphocyte mediated immunity | 29 | 2.94 | 4.26E-03 |
| 0006643 | membrane lipid metabolic process | 36 | 2.94 | 1.93E-04 |
| 0030198 | extracellular matrix organization | 56 | 2.86 | 7.73E-08 |
| 0043062 | extracellular structure organization | 56 | 2.85 | 8.71E-08 |
