# Supplementary material for: Expression profiling of long noncoding RNA identifies lnc‐MMP3‐1 as a prognostic biomarker in external auditory canal squamous cell carcinoma
Source: Cancer Med. 2017 Sep 29;6(11):2541–51. doi: 10.1002/cam4.1213 (PMC5673923; doi:10.1002/cam4.1213)
Supplement: Supplementary file 5 — Table S4. Cellular components enrichment analyses of differential expression genes. [file CAM4-6-2541-s005.doc]

SuppInfo Table 4. Cellular components enrichment analyses of differential expression genes

| GO ID | Term | Gene | Fold Enrichment | P value |
| --- | --- | --- | --- | --- |
| 0001533 | cornified envelope | 20 | 6.77 | 7.40E-08 |
| 0042470 | melanosome | 27 | 4.05 | 2.94E-06 |
| 0048770 | pigment granule | 27 | 4.05 | 2.94E-06 |
| 0005604 | basement membrane | 21 | 3.38 | 3.02E-03 |
| 0044420 | extracellular matrix component | 25 | 3.04 | 2.24E-03 |
| 0043209 | myelin sheath | 28 | 2.48 | 2.38E-02 |
| 0005788 | endoplasmic reticulum lumen | 31 | 2.44 | 1.13E-02 |
| 0005578 | proteinaceous extracellular matrix | 52 | 2.27 | 1.24E-04 |
| 0031012 | extracellular matrix | 74 | 2.26 | 3.04E-07 |
| 0000323 | lytic vacuole | 75 | 2.16 | 1.53E-06 |
| 0005764 | lysosome | 75 | 2.16 | 1.53E-06 |
| 0030055 | cell-substrate junction | 53 | 2.11 | 8.25E-04 |
| 0005925 | focal adhesion | 51 | 2.06 | 2.39E-03 |
| 0005924 | cell-substrate adherens junction | 51 | 2.05 | 2.94E-03 |
| 0005913 | cell-cell adherens junction | 41 | 2.04 | 3.13E-02 |
| 0098552 | side of membrane | 56 | 1.98 | 2.47E-03 |
| 0044432 | endoplasmic reticulum part | 148 | 1.98 | 6.45E-12 |
| 0009986 | cell surface | 97 | 1.98 | 4.73E-07 |
| 0070062 | extracellular exosome | 340 | 1.98 | 2.54E-32 |
| 0070161 | anchoring junction | 87 | 1.97 | 4.89E-06 |
