# Supplementary material for: Expression profiling of long noncoding RNA identifies lnc‐MMP3‐1 as a prognostic biomarker in external auditory canal squamous cell carcinoma
Source: Cancer Med. 2017 Sep 29;6(11):2541–51. doi: 10.1002/cam4.1213 (PMC5673923; doi:10.1002/cam4.1213)
Supplement: Supplementary file 6 — Table S5. Molecular functions enrichment analyses of differential expression genes. [file CAM4-6-2541-s006.doc]

**SuppInfo Table 5**. Molecular functions enrichment analyses of differential

expression genes

| GO ID | Term | Gene | Fold Enrichment | P value |
| --- | --- | --- | --- | --- |
| 0001948 | glycoprotein binding | 21 | 3.07 | 2.62E-02 |
| 0098631 | protein binding involved in cell adhesion | 39 | 2.13 | 4.15E-02 |
| 0050839 | cell adhesion molecule binding | 59 | 2.06 | 8.62E-04 |
| 0032403 | protein complex binding | 91 | 1.79 | 3.49E-04 |
| 0005102 | receptor binding | 165 | 1.71 | 6.33E-08 |
| 0019899 | enzyme binding | 176 | 1.53 | 4.57E-05 |
| 0044877 | macromolecular complex binding | 127 | 1.52 | 7.87E-03 |
| 0003824 | catalytic activity | 478 | 1.26 | 5.91E-06 |
| 0005515 | protein binding | 846 | 1.23 | 6.33E-16 |
| 0005488 | binding | 1034 | 1.14 | 7.13E-12 |
| 0003674 | molecular_function | 1190 | 1.11 | 6.85E-15 |
| - | Unclassified | 128 | 0.53 | 0.00E+00 |
| 0004930 | G-protein coupled receptor activity | 27 | 0.49 | 3.17E-02 |
| 0004984 | olfactory receptor activity | 2 | < 0.2 | 2.43E-06 |
