# Supplementary material for: Expression profiling of long noncoding RNA identifies lnc‐MMP3‐1 as a prognostic biomarker in external auditory canal squamous cell carcinoma
Source: Cancer Med. 2017 Sep 29;6(11):2541–51. doi: 10.1002/cam4.1213 (PMC5673923; doi:10.1002/cam4.1213)
Supplement: Supplementary file 7 — Table S6. KEGG enrichment analyses of differential expression genes. [file CAM4-6-2541-s007.doc]

**SuppInfo Table 6**. KEGG enrichment analyses of differential expression genes

| Term | Gene | P value | Fold Enrichment | FDR |
| --- | --- | --- | --- | --- |
| Phagosome | 28 | 2.70E-08 | 3.4 | 3.60E-05 |
| Protein processing in endoplasmic reticulum | 25 | 9.20E-06 | 2.8 | 1.20E-02 |
| Rheumatoid arthritis | 17 | 1.30E-05 | 3.6 | 1.70E-02 |
| Lysosome | 20 | 1.90E-05 | 3.1 | 2.50E-02 |
| ECM-receptor interaction | 16 | 4.80E-05 | 3.4 | 6.20E-02 |
| Focal adhesion | 25 | 2.40E-04 | 2.3 | 3.10E-01 |
| Prion diseases | 9 | 2.60E-04 | 5.1 | 3.40E-01 |
| Vibrio cholerae infection | 11 | 4.10E-04 | 3.9 | 5.40E-01 |
| Osteoclast differentiation | 18 | 5.60E-04 | 2.6 | 7.30E-01 |
| Amoebiasis | 15 | 1.40E-03 | 2.6 | 1.80E+00 |
| Toll-like receptor signaling pathway | 15 | 1.40E-03 | 2.6 | 1.80E+00 |
| Regulation of actin cytoskeleton | 23 | 1.90E-03 | 2 | 2.40E+00 |
| Proteoglycans in cancer | 21 | 4.80E-03 | 2 | 6.10E+00 |
| PI3K-Akt signaling pathway | 31 | 5.30E-03 | 1.7 | 6.70E+00 |
| Natural killer cell mediated cytotoxicity | 15 | 5.30E-03 | 2.3 | 6.70E+00 |
| Cell cycle | 15 | 6.10E-03 | 2.3 | 7.70E+00 |
| Protein export | 6 | 6.40E-03 | 4.9 | 8.00E+00 |
| Staphylococcus aureus infection | 9 | 7.40E-03 | 3.1 | 9.20E+00 |
| Proteasome | 8 | 8.10E-03 | 3.4 | 1.00E+01 |
| Chemokine signaling pathway | 19 | 1.00E-02 | 1.9 | 1.30E+01 |
