# Supplementary material for: Expression profiling of long noncoding RNA identifies lnc‐MMP3‐1 as a prognostic biomarker in external auditory canal squamous cell carcinoma
Source: Cancer Med. 2017 Sep 29;6(11):2541–51. doi: 10.1002/cam4.1213 (PMC5673923; doi:10.1002/cam4.1213)
Supplement: Supplementary file 8 — Table S7. Treatment modality of EAC SCC patients. [file CAM4-6-2541-s008.doc]

**SuppInfo Table 7. Treatment modality of EAC SCC patients**.

| TNM stage | Surgery alone | Surgery +  adjuvant radiotherapy | Primary surgery + salvage surgery | Surgery + adjuvant chemo- radiotherapy | Total |
| --- | --- | --- | --- | --- | --- |
| I stage | 0 | 1 | 0 | 0 | 1 |
| II stage | 8 | 1 | 0 | 0 | 9 |
| III stage | 4 | 4 | 1 | 1 | 10 |
| IV stage | 7 | 8 | 3 | 5 | 23 |
| Total | 19 | 14 | 4 | 6 | 43 |
